# A quantitative determination of lipid bilayer deposition efficiency using AFM†

**DOI:** 10.1039/d1ra01920a

**Published:** 2021-06-02

**Authors:** Mary H. Wood, David C. Milan, Richard J. Nichols, Michael T. L. Casford, Sarah L. Horswell

**Affiliations:** School of Chemistry, University of Birmingham Birmingham B15 2TT UK s.l.horswell@bham.ac.uk mary.wood@epfl.ch; Department of Chemistry, University of Liverpool Liverpool L69 7ZD UK; Department of Chemistry, University of Cambridge Lensfield Road Cambridge CB2 1EW UK

## Abstract

The efficacy of a number of different methods for depositing a dimyristoylphosphatidylcholine (DMPC) lipid bilayer or DMPC–cholesterol (3 : 1) mixed bilayer onto a silicon substrate has been investigated in a quantitative manner using atomic force microscopy (AFM) image analysis to extract surface coverage. Complementary AFM-IR measurements were used to confirm the presence of the lipids. For the Langmuir–Blodgett/Schaefer deposition method at temperatures below the chain-melting transition temperature (*T*_m_), a large number of bilayer defects resulted when DMPC was deposited from a water subphase. Addition of calcium ions to the trough led to smaller, more frequent defects, whereas addition of cholesterol to the lipid mixture led to a vast improvement in bilayer coverage. Poor coverage was achieved for deposition at temperatures above *T*_m_. Formation of the deposited bilayer from vesicle fusion proved a more reliable method for all systems, with formation of near-complete bilayers within 60 seconds at temperatures above *T*_m_, although this method led to a higher probability of multilayer formation and rougher bilayer surfaces.

## Abstract 2

The efficacy of different methods for depositing a DMPC or mixed DMPC–cholesterol (3 : 1) lipid bilayer onto a silicon substrate has been investigated in a quantitative manner using atomic force microscopy image analysis to extract surface coverage.

## Graphical abstract

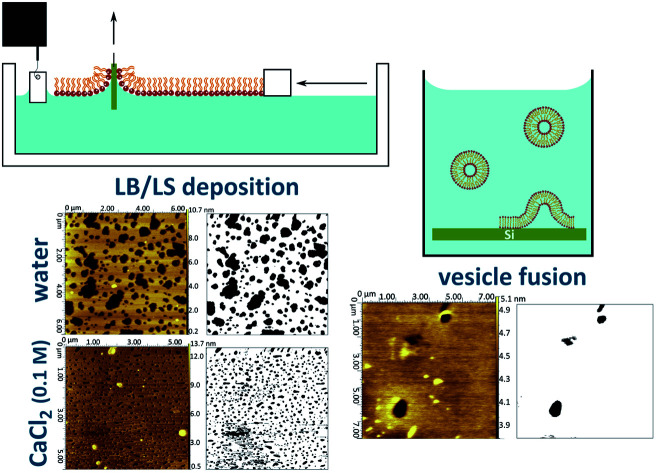

## Introduction

Solid-supported lipid bilayers are widely used for studying fragile model cell membrane structures using techniques such as neutron reflectometry,^1–5^ X-ray photoelectron spectroscopy,^6^ quartz crystal microbalance methods,^7–9^ surface-based vibrational spectroscopic techniques,^10–15^ atomic force microscopy^16–19^ (AFM) and electrochemical measurements,^20–23^ amongst others. However, for some systems it can be difficult to achieve full bilayer coverage, with patches of the substrate surface remaining exposed. This can be disadvantageous when attempting to extract values for parameters such as the bilayer resistance or capacitance. A variety of different deposition techniques are described in the literature; here, we compare a range of methods for depositing a 1,2-dimyristoyl-*sn*-glycero-3-phosphocholine (dimyristoyl phosphatidyl choline, DMPC) bilayer or DMPC–cholesterol (3 : 1) mixed bilayer onto a silicon surface.

DMPC is a commonly-studied zwitterionic phospholipid that is a key component of pulmonary surfactant mixtures found on the surfaces of alveoli^24,25^ and also provides a useful model for the structural phosphatidylcholines ubiquitous to eukaryotic cell membranes.^26^ Cholesterol, which is present in many natural cell membranes (for example, constituting approximately 20% of a mammalian red blood cell membrane^27^), is known to moderate the fluidity of lipid bilayers and may also have a condensing effect on the phase.^28^ A thicker film is also often seen for deposited bilayers of DMPC that include cholesterol than for pure DMPC bilayers, arising from more efficient headgroup packing and hence a lower chain tilt angle.^29^

The Langmuir–Blodgett/Langmuir–Schaefer (LB/LS) technique is a well-established method for deposition of mono- or multilayers of surfactants onto solid substrates.^30^ Briefly, it involves placing the substrate in an aqueous subphase, at an orientation perpendicular to the interface, and raising it from the subphase through a monolayer of the surfactant at the air–water interface so that the monolayer is transferred to the substrate. The substrate is then lowered to the interface horizontally in order to pick up a second layer of surfactant and form a bilayer. Subsequent dipping and raising of the substrate will lead to an increasing number of deposited layers. The high level of control afforded by this technique with regards to the number of layers and the molecular area (*via* precise measurement of the surface pressure) has ensured its universal adoption with little alteration for the past century. Another common method of lipid bilayer formation on a solid support is *via* liposome fusion, whereby the surface is exposed to a suspension of phospholipid vesicles; as the liposomes encounter the surface, their outer membranes may deform and eventually rupture to form the surface bilayer. Vesicles have also been observed to adsorb whilst still intact and to coalesce over time to form islands on the surface. The exact kinetics of bilayer formation are system-dependent but in general, complete bilayer formation has been reported to occur within 1–2 hours.^7^ A so-called “hybrid” deposition method has also been advocated, in which the first layer is deposited *via* the LB/LS method and the second *via* vesicle fusion. This is particularly pertinent for instances where asymmetric bilayers are required but may also have some advantages in terms of ensuring optimum coverage.^31,32^ Divalent ions such as calcium are known to significantly affect bilayer formation, both on external substrates and *in vivo* by means of vesicle fusion.^33,34^

In this work, we present a quantitative approach for determining the coverage extent for lipid bilayers formed on silicon surfaces using AFM and on gold using AFM-IR (atomic force microscopy-infrared). Several of the methods discussed above are compared and the coverages reported as a percentage value in each case. Although the surface is referred to as silicon throughout, it is worth noting that a native silicon dioxide layer of the order 1–2 nm thickness will form naturally and so the surface may be thought more properly as silica. AFM-IR is a powerful technique that allows infrared spectroscopy mapping at a much greater spatial resolution than is usual for standard IR spectra; this is obtained by using the AFM tip to monitor thermal expansion caused by the incident infrared beam when a molecular vibration is excited at the surface.^35^

## Experimental

### Materials

Silicon wafers (p-type, {100}, 1 mm thickness) were obtained from MicroChemicals GmbH and cleaned with acidic piranha solution (30% H_2_O_2_ and concentrated H_2_SO_4_ in a 1 : 3 ratio) before use (**Caution**: acidic piranha solution may react with organic compounds to form an explosive mixture). Gold (111)-coated slides were acquired from Arrandee and cleaned with ethanol before use. 1,2,dimyristoyl-*sn*-glycero-3-phosphocholine (DMPC) and cholesterol were obtained from Avanti Polar Lipids, Inc. Lipid solutions were prepared in chloroform or chloroform : methanol (9 : 1); both solvents were HPLC grade and obtained from Sigma-Aldrich. Calcium chloride was obtained from Sigma Aldrich (purity > 99.9%) and stored under argon and potassium chloride was obtained from Alfa Aesar (purity 99%). Water purified with a tandem Milli-Q Gradient A10 system (Millipore, France, resistivity 18 MΩ cm, TOC < 5 ppb) was used throughout.

### Surface pressure isotherms

A Langmuir trough (NIMA) was used for both surface pressure isotherms and LB/LS depositions. The trough was cleaned with chloroform before being filled with ultrapure water (UPW) or other aqueous subphase. The temperature of the subphase was maintained with a thermostatted recirculating water bath and the cleanliness of the water surface was verified prior to the addition of the lipid solution (2 mg mL^−1^, 60 μL) using a microlitre syringe. The solvent was allowed to evaporate and the monolayer compressed until collapse. Isotherms were measured at 16 °C and 29 °C, below and above the chain-melting transition, respectively. The estimated error in temperature is ±1 °C and the estimated error in the area per molecule is ±2–3 Å^2^.

### Lipid deposition

#### LB/LS method

The substrates were submerged using a custom-made holder in the Langmuir trough prior to the addition of the lipid to the subphase surface. The monolayer was compressed to a pressure of 40 mN m^−1^ and the substrates drawn slowly (1.1 mm min^−1^) up through the layer (Langmuir–Blodgett deposition). To complete the bilayer, the substrates were dried in argon and then brought down to the water surface again until just touching (Langmuir–Schaefer deposition). They were then carefully removed and dried in argon again. For the calcium study, the trough was filled with an aqueous CaCl_2_ solution (0.1 M) instead of UPW. Lipids were deposited at 16 °C or 28 °C (below and above the chain-melting transition, respectively).

#### Vesicles method

A 2 mL lipid solution (1 mg mL^−1^) in chloroform was added to a test tube and agitated under argon until the solvent had evaporated, to leave a dried lipid film that was well spread across the base of the tube. 2 mL of either pure H_2_O or 0.1 M CaCl_2_ solution were then added and the solution sonicated in a Bransonic M1800H-E ultrasonic bath (70 W, 40 kHz) at >30 °C for 2 h. Dynamic light scattering (DLS) measurements using a Malvern Panalytical Zetasizer instrument (*λ* = 633 nm, measurement angle 90°) were used to confirm the size of the final vesicles (*Z*-average: 100–150 nm, examples shown in Fig. S1†). To deposit the bilayer, the silicon pieces were immersed in the vesicle solution for the required amount of time, then gently rinsed with UPW and dried under argon. Lipids were deposited at 18 °C or 28 °C (below and above the chain-melting transition, respectively, unless stated otherwise).

### AFM

AFM images were measured using a 5500 Agilent Scanning Probe Microscope and a Keysight 9500 AFM control unit with an Agilent N9410S AFM scanner. For analysis of the lipid bilayer coverage, samples were measured in air in non-contact (AC) mode using silicon tips mounted on cantilevers with force constants of 10–130 N m^−1^ at scanning rates of 1 line per s. Images were flattened and plane-fitted as required. Height profiles across the entire image were used to determine a suitable height threshold value above which it could be assumed the surface was covered entirely in lipid and below which the substrate was covered only partially or bare. The image was then converted to a binarised format and the number of pixels above the threshold value counted to give a % coverage. Image analysis was conducted using the software Gwyddion 2.54, MATLAB 2018b and ImageJ 1.51. At least 10 images were collected across at least two samples for each system in order to ensure the average obtained was suitably representative. Samples were analysed as soon as possible after the deposition process, to minimise any possible effect of change over time. A few samples were re-analysed after some months (with no special storage) and showed no change in overall average surface coverage.

### AFM-IR

AFM-IR measurements were conducted on a nanoIR2 spectrometer (Anasys Instruments). Spectra were acquired in contact mode. IR images were collected by setting the laser to 1738 cm^−1^ and plotting the intensity variation across the AFM scan.

## Results and discussion

### Surface pressure isotherms

**Fig. 1:**
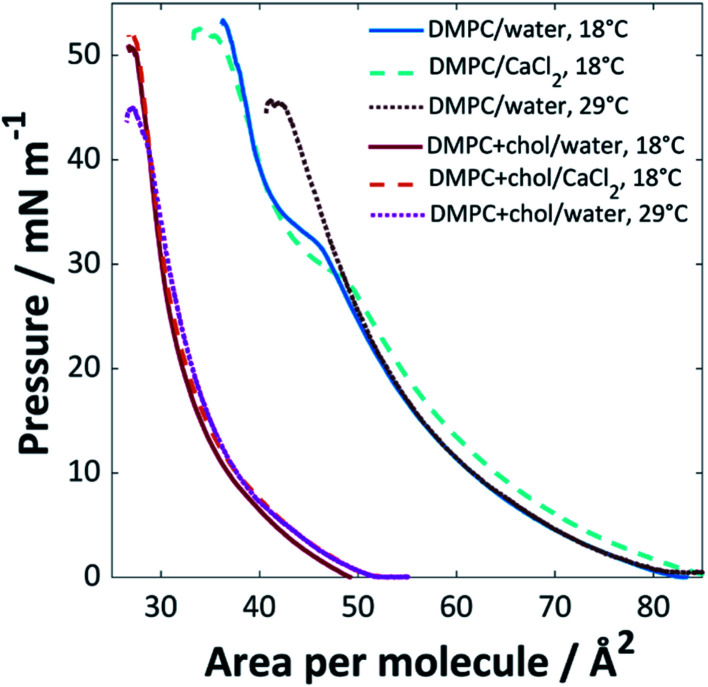
Surface pressure isotherms for pure DMPC and DMPC : cholesterol (3 : 1) mixtures on UPW or CaCl_2_ (0.1 M) solution at 18 °C or 29 °C (±1 °C).

The surface pressure isotherms for DMPC and DMPC : cholesterol (3 : 1) mixtures on UPW or calcium chloride solution (0.1 M) at 18 °C or 29 °C are shown in Fig. 1. The monolayer of pure DMPC at 18 °C collapsed at 53 mN m^−1^ at an area per lipid molecule of ∼37 Å^2^ and extrapolation of the steeper portion of the curve to the abscissa gives a limiting area of 49 Å^2^, in good agreement with previously reported values^36,37^ (lipids with the phosphocholine headgroup form monolayers with a minimum molecular area that is determined by the packing of the relatively bulky headgroups, such that the hydrocarbon tails are generally tilted away from the surface normal^38^). A phase transition was observed around 32 mN m^−1^, corresponding to the transition between the liquid expanded and liquid condensed phases.^39^

The addition of cholesterol to the DMPC lipid solution (3 : 1 DMPC : cholesterol) significantly decreased the area per molecule, to ∼27 Å^2^ at the point of collapse, with a limiting area of 34 Å^2^; this, and the loss of the expanded-condensed phase transition, indicates that the cholesterol causes a change in packing structure. This phenomenon is well-documented; Róg *et al.* found from simulations that addition of 22% cholesterol to DMPC is expected to reduce the area per molecule from 60 to 53 Å^2^ at 37 °C, with a significantly reduced tilt angle for the alkyl chains in the DMPC–cholesterol bilayer compared with those of the pure DMPC bilayer.^40^ The decreased area per molecule for lipid/cholesterol mixtures is believed to arise from a more efficient packing structure, with the long hydrocarbon chains of the lipid able to wrap around the smaller cholesterol molecules, also known as a ‘molecular cavity effect’.^41,42^ In monolayers, cholesterol has been found to disorder liquid condensed phases and order liquid expanded phases, which may result in the loss of the phase transition.^43^

When CaCl_2_ was used as the subphase, a decrease in pressure for the expanded phase-condensed phase transition was observed. This effect has been observed for divalent cations; the lipid's polar headgroup is known to be at least partially hydrated, giving rise to an interlayer between the bulk water and aliphatic tail group region. The calcium cations are able to permeate this interlayer and interact strongly with the polar headgroups to favour the formation of the well-packed gel state at lower pressures.^44,45^ This is believed to arise from an inner-sphere complex formed between the calcium ions and negatively-charged phosphate groups in the PC headgroups, which leads to partial dehydration and restructuring of the phosphate. Calcium is one of the most effective cations in this regard, mainly because of its size, as reflected by the relatively high ion-lipid binding constant^46^ (chloride ions are also known to affect the monolayer packing by preferentially adsorbing to the ammonium group, resulting in increased lipid–lipid repulsion and hence an increased molecular area; however, this effect is clearly less strongly felt than that of the calcium cations^46,47^).

The chain-melting temperature (*T*_m_) for DMPC is 24 °C.^48^ Little change was seen for the DMPC : cholesterol (3 : 1) mixture when the isotherm was measured at 29 °C, other than a lower pressure of collapse, suggesting a slightly less stable monolayer. Above ∼30 mol% cholesterol content, DMPC is expected to occupy a liquid-ordered phase below and above *T*_m_ ^28^ and the isotherm remains therefore mainly unaffected by the change in temperature. For the pure DMPC system, a loss of the expanded-condensed phase transition was observed at 29 °C; at this increased temperature the lipids are expected to be in the liquid crystalline phase and therefore a much greater pressure would be required to force the monolayer into the condensed phase at the air/water interface than the range applied here.

### Depositions on silicon

#### Langmuir–Blodgett/Langmuir–Schaefer method

**Fig. 2:**
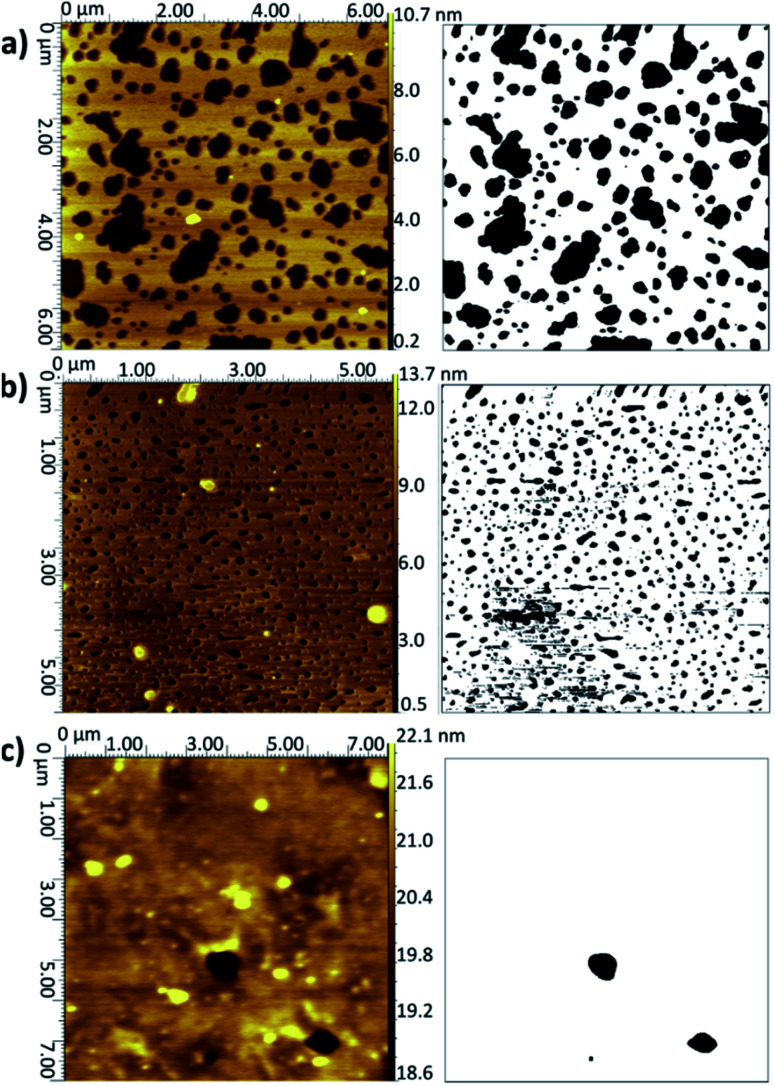
AFM topographical images (left) and corresponding binarised data (right) for LB/LS bilayers deposited onto silicon at 16 °C: (a) DMPC from UPW, (b) DMPC from CaCl_2_ (0.1 M) solution, (c) DMPC : cholesterol (3 : 1) from UPW. (Note slightly different xy scales for the three images.) Further examples are given in Fig. S2–S4 of the ESI.†

The average surface coverages and lipid bilayer thicknesses for the LB/LS samples are summarised in Table 1 and selected representative examples of the images are shown in Fig. 2 (with further examples in Fig. S2–S4).† Koenig *et al.* report coverage values for DPPC on silica as around 70% (±20%) using a combination of AFM and neutron reflectometry,^5^ in good agreement with the value seen here for DMPC on silicon. As can be seen in Fig. 2a, the LB/LS bilayers deposited for pure DMPC on silicon have a large number of defects uniformly distributed across the surface. Phosphatidylcholines have been reported as less strongly adhering to solid supports than other lipids.^49^ Infrared measurements have previously shown them to interact more with water molecules, *via* hydrogen bonding, than with other lipid molecules, in contrast to some other lipids such as phosphatidylethanolamines.^50^

**Table 1:** Summary of coverage parameters for bilayers deposited onto silicon *via* the LB/LS method as determined by AFM (*T* is the deposition temperature. Quoted errors are the data standard deviations)

Lipid system	Subphase	*T*/°C	Average coverage/%	Average defect thickness/nm
DMPC	H_2_O	16	72 (±11)	4.5 (±0.5)
DMPC	H_2_O	28	6 (±6)	4.7 (±0.3)
DMPC	CaCl_2_ (0.1 M)	16	85 (±5)	4.6 (±0.7)
DMPC : cholesterol (3 : 1)	H_2_O	16	97 (±2)	4.7 (±1.3)
DMPC : cholesterol (3 : 1)	H_2_O	28	33 (±28)	4.8 (±0.6)
DMPC : cholesterol (3 : 1)	CaCl_2_ (0.1 M)	16	97 (±2)	4.9 (±1.7)

When a calcium chloride solution is used in the Langmuir trough rather than UPW, a better overall coverage is seen and the defects are generally smaller in diameter, approximately 0.2 μm compared with 1 μm for the UPW subphase (Fig. 2b). This may arise from the better packing of the lipid monolayers effected by the bridging calcium cations, as also seen in the surface pressure isotherms. Breakthrough forces for DMPC bilayers on mica have been shown to increase when a monovalent cation (Na^+^) is introduced and further still for a divalent cation (Mg^2+^).^51^ This would suggest that the lipid bilayers are more tightly packed and hence harder to disrupt. As mentioned above, this may arise from interactions between the calcium ions and negatively-charged phosphate groups. Furthermore, molecular dynamics simulations for POPC bilayers in salt solutions have demonstrated that the binding of Ca^2+^ cations to the lipid carbonyl oxygen atoms (4.2 for each cation) results in the formation of large and cumbersome lipid–ion complexes,^52^ which subsequently limits lipid self-diffusion and rotational diffusion; the lipids are hence less likely to rearrange or diffuse on the surface to allow larger defects to form.

Addition of cholesterol to the DMPC bilayer leads to a significant improvement in coverage, with near-full coverage seen for both UPW and the calcium chloride solution; the cholesterol permits better packing of the bilayer and hence fewer defects arise. For DMPC/cholesterol mixtures, it has been suggested that depositing at a temperature of 28 °C may improve the homogeneity of the deposited bilayer.^10^ However, for both the pure DMPC bilayer and DMPC/cholesterol mixture deposited using the LB/LS technique here, a considerable decrease in coverage is seen when the temperature is raised from 16 °C to 28 °C. This is unsurprising for the pure DMPC; at the higher temperature, the lipids will be in the liquid crystalline phase and hence less able to pack together efficiently on the surface to form a defectless bilayer. Similarly, Morigaki *et al.* report a much greater number of large defects for bilayers of DiynePC (diacetylene phospholipid (1,2-bis(10,12-tricosadiynoyl)-*sn*-3-phosphocholine)) deposited at 28 °C using the LB/LS method compared with those deposited at 16 °C (above and below the DiynePC triple point temperature of 20 °C, respectively). They attribute this to differences in the domain sizes resulting from a different phase transition route.^53^ For the DMPC/cholesterol mixture, the lower collapse pressure seen for the isotherm indicates the monolayer is less stable at this elevated temperature and therefore may not transfer to the surface in such an ordered fashion; the high standard deviation for the coverage value reflects the patchiness observed across the samples.

### Thickness measurements

To determine the layer thickness values reported in Table 1, a large number of height profiles were taken across each AFM image, the background was subtracted and the depths of each defect were recorded to give an overall average. Whilst this method was simple, it was hampered by a large error margin caused by the roughnesses of the height profiles and difficulty in judging exactly when and if the AFM tip had reached the underlying substrate or whether there remained surface contamination at the base (examples shown in Fig. S5†). There is no obvious difference within error between the bilayer thicknesses for each of the DMPC samples and the values are within the range of thicknesses reported in the literature.^54–57^

**Fig. 3:**
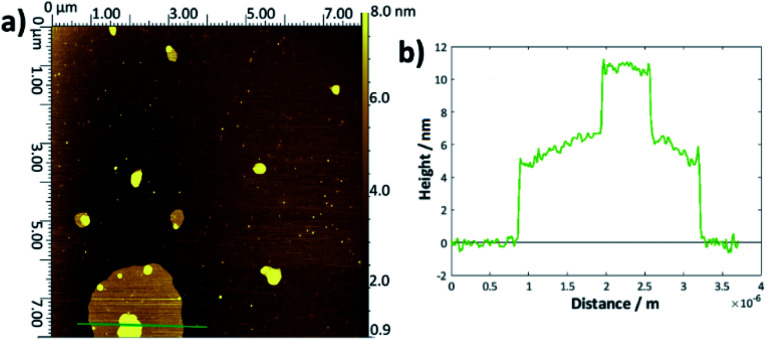
(a) Example AFM topological image for DMPC:cholesterol deposited onto silicon at 28 °C *via* the LB/LS technique, showing an island multilayer, (b) height profile taken across the green line shown in (a).

There may be a slight increase in the average thickness for the DMPC:cholesterol results, which would accord with the lower area per molecule observed in the surface–pressure isotherms, since a higher chain tilt angle would be expected for a more compressed layer.^29,58^ However, the increase in standard deviation reflects the greater range of values obtained for these systems; indeed, for several samples, it seemed likely that there may have been multilayer formation as the defects were of the order 8–15 nm deep. For the thickness measurements quoted, any values greater than 7 nm were discounted as outliers. The upper surface of the DMPC:cholesterol bi/multilayers were also generally rougher than for the pure DMPC. For the higher temperature DMPC:cholesterol samples deposited at 28 °C, the height profiles frequently showed double bilayers/multilayers—an example is shown in Fig. 3. This may arise from the relative instability of the bilayers formed at this higher temperature, leading to buckling or folding in the Langmuir trough and hence multilayer deposition. Whilst generally the measured thicknesses lie within the range of literature-reported values^54–58^ and appear reasonable, their main purpose has been to allow for the confirmation of bilayer deposition and hence to allow determination of surface coverage.

#### Vesicles Method

**Fig. 4:**
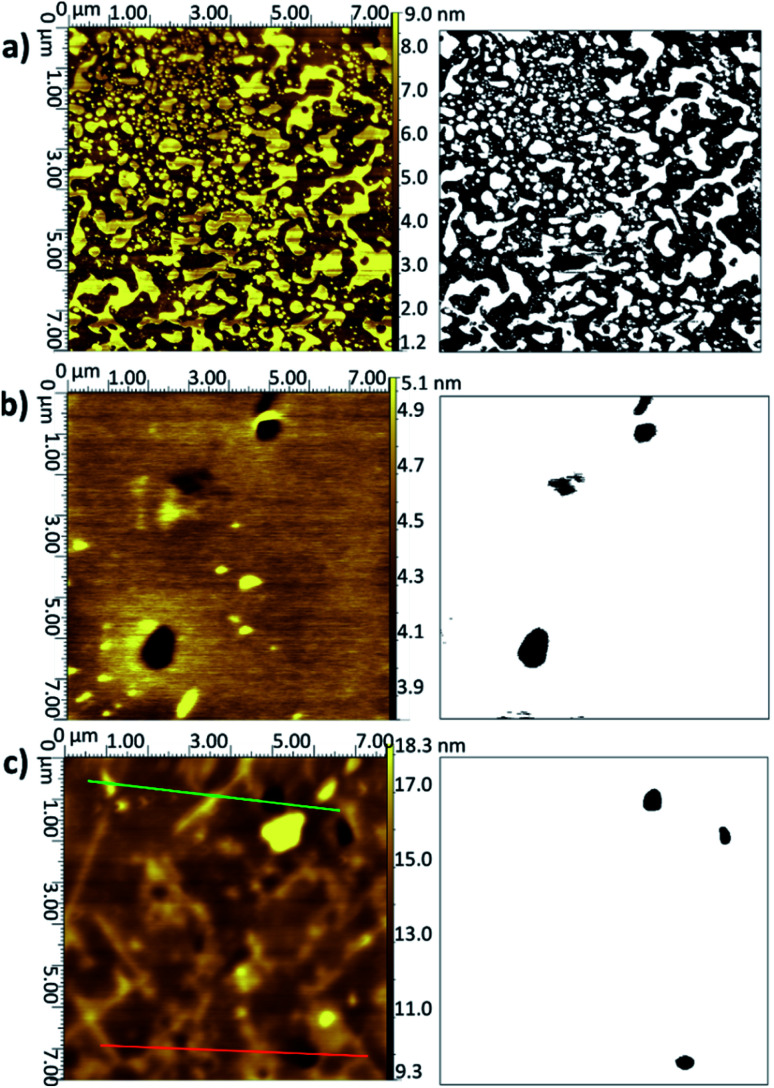
AFM topographical images (left) and corresponding binarised data (right) for layers on Si deposited from vesicles of DMPC in UPW (sonicated at 28 °C) with the substrate removed after (a) 30 s, (b) 60 s, (c) 7200 s.

**Fig. 5:**
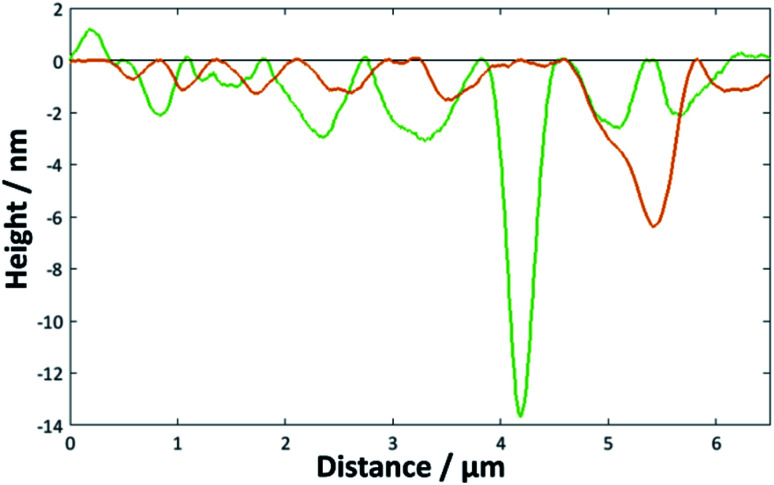
Height profiles for the image shown in Fig. 4c across the marked green and orange lines.

The surface coverages and associated thicknesses for the silicon samples exposed to vesicle solutions are summarised in Table 2. It is clear that for the DMPC bilayers, the vesicle fusion is a much better method for forming a near-full coating of the silicon surface in a very short time. Example images for the DMPC/H_2_O vesicle samples are shown in Fig. 4 (further examples for all systems listed in Table 2 are given in Fig. S6 to S25 in the ESI†). After 30 s (Fig. 4a), roughly half the surface is covered; the AFM topographical images show clusters of islands formed by the fusing vesicles. By 60 s, the surface is essentially covered bar a few small defects and there is no real change upon exposure for longer time periods. After 2 h, the surface has become noticeably rougher, as demonstrated in the height profiles shown in Fig. 5. There was also a greater number of deeper defects (>7 nm), suggesting that over time multilayers had formed over the initial bilayer—an average value for these thicker defects is also given in the table, although the high standard deviation is indicative of varying numbers of deposited lipid layers. Anderson *et al.* report a stronger adhesion of DPPC bilayers deposited using the LB/LS method than for those adsorbed *via* vesicle fusion, as reflected in the repulsion forces measured by surface force apparatus (SFA). They attribute this to lower undulation forces in the LB/LS bilayers (arising from higher tension).^59^ The higher undulation may be the cause here of the increased roughness at longer exposure times. In some instances, roughly circular features with a height much greater than that expected for a bilayer, but lower than the diameter of the vesicles (100–150 nm), were observed across the surface. Attwood *et al.* observe similar features for DPPC vesicles on mica surfaces; after recording AFM images of very dilute solutions of the lipid vesicles on the surface, they attribute these to partially fused vesicles.^16^ Given the size of the features, it would seem likely that the same explanation may be accorded in this case.

**Table 2:** Summary of coverage parameters for bilayers deposited onto silicon from vesicle fusion as determined by AFM (*T* is the temperature of deposition. Quoted errors are the data standard deviations)

Lipid system	Subphase	Time/s	*T*/°C	Average coverage/%	Average defect thickness/nm
DMPC	H_2_O	30	28	56 (±19)	4.7 (±0.4)
		60		98 (±1)	4.6 (±0.9)
		300		99.1 (±0.6)	4.6 (±1.0)
		1800		98.1 (±0.6)	4.3 (±0.7)
		7200		95 (±4)	4.8 (±0.9)
					8.3 (±2.2)
DMPC	CaCl_2_	30	28	99 (±1)	4.7 (±0.5)
		60		99.5 (±0.2)	4.5 (±0.6)
		120		99.4 (±0.3)	4.7 (±0.7)
		1800		99.2 (±0.7)	4.9 (±0.8)
		7200		99.8 (±0.1)	4.6 (±0.5)
DMPC : cholesterol	H_2_O	30	28	95 (±1)	4.5 (±0.5)
					8.5 (±1.3)
		60		98.4 (±0.8)	4.6 (±0.6)
					8.0 (±1.5)
		120		99 (±1)	4.5 (±0.4)
					8.6 (±2.0)
		600		98 (±2)	4.6 (±0.6)
					7.3 (±0.9)
		1200		98 (±2)	4.6 (±0.5)
					7.7 (±1.0)
		1800		99.3 (±0.4)	4.5 (±0.4)
					8.7 (±2.8)
		7200		99.7 (±0.3)	5.1 (±0.5)
					8.2 (±2.7)
DMPC	H_2_O	60	18	13 (±8)	4.6 (±0.4)
		300		20 (±10)	4.8 (±0.4)
		1800		99 (±1)	4.8 (±0.6)

When the temperature was lowered below *T*_m_ to 18 °C, it took much longer for the full bilayer to form on the surface, with only around 20% coverage seen after 5 min of sonicating in the lipid vesicle solution. By 30 min, the surface was essentially fully covered. Reimhult *et al.* observed a similar temperature-dependent effect for egg-yolk PC vesicle fusion on SiO_2_: at lower temperatures, it took longer for intact vesicles adsorbed on the surface to rupture and form the bilayer. At 0 °C (measured using a non-freezing buffer), they saw purely adsorption of nonruptured vesicles with no fusion at all, demonstrating the importance of temperature-dependent lateral interactions between the adsorbed vesicles.^60^ However, Lind *et al.* saw a lower overall coverage for DPPC bilayers formed on silicon from vesicles at 50 °C—the *T*_m_ of DPPC being around 41.5 °C—and then cooled to 25 °C compared with those formed at 25 °C; they attributed this to a greater area per lipid at the higher temperature, leading to subsequent shrinking of the bilayer upon cooling.^61^ This may, therefore, indicate that the deposition method could be optimised by starting at a higher temperature to initiate rupture but then cooling to ensure the best possible coverage. However, in this work, a suitably high coverage is seen at temperatures > *T*_m_.

Little difference is seen for the vesicles suspended in a CaCl_2_ solution or when cholesterol was added, with high (>90%) coverage also seen within a very short time. For these two systems, the bilayer coverage was essentially completed within 30 s, indicating that there may be some small advantage incurred by the addition of the salt and/or cholesterol. However, the difference is considerably less marked in comparison with the LB/LS results. In addition, there is a higher proportional error in time measurement for these <1 min samples, reflected in the higher standard deviations recorded. The rôle of cholesterol in vesicle fusion is less unequivocal than for LB/LS depositions—whilst it has been reported to aid fusion for DMPC onto gold substrates,^29^ in other systems it has been found to stabilise hemifusion and hence prevent formation of a complete bilayer.^62^ As noted in Table 2, in several of the cholesterol samples two ranges of defect thickness were observed; these were divided into those below and above 6 nm, and both average values recorded. It is possible that the thicker values reflect either multilayer formation or incomplete fusion on the surface.

The effects of a calcium-containing solution on vesicle fusion depend on the exact balance of charges in each system. Whereas for the LB/LS method the calcium ion simply acted to ensure better packing of the lipid monolayer in the Langmuir trough and hence better deposition, adding calcium may actually stabilise vesicles comprising polar lipids in solution and thus hinder their adsorption onto the surface.^7^ Conversely, when the vesicles have adsorbed, the salt solution may increase likelihood of rupture by permitting stronger vesicle–vesicle interactions.^63,64^ Here, there is a possible slight improvement in the rate of forming a full bilayer of DMPC when the calcium is introduced, although as the bilayer formation was also extremely fast for samples prepared using calcium-free solution, the effect is relatively small. The vesicles' high curvature in the solution phase makes them thermodynamically unstable with respect to fusion onto the surface to form extended bilayers, particularly for smaller vesicles comprising only one lipid type, as evidenced in this work^65^ (it is worth noting that the results reported here for silicon (or SiO_2_) may not relate to other surfaces as vesicle fusion has been shown to be highly material-dependent^7,9,16,66^).

Whereas the LB/LS bilayers are relatively easy to assess using AFM because of the clarity and frequency of the defects, this was not always the case for the samples prepared by vesicle fusion. In some instances, it was difficult to judge between a full bilayer and a blank substrate; this was further complicated by the ability of vesicles to adhere occasionally to a full bilayer, despite rinsing to remove excess lipids.^61^ To remove these sources of confusion, AFM-IR was used to give simultaneous spectroscopic maps of the AFM image and hence discern between lipid and bare substrate. Gold substrates were used for AFM-IR because the technique is unable to give a strong enough signal from the silicon. There are some subtle differences in bilayer structure on gold and silicon/silica-based substrates (for example, the tilt angle of the chains is a little higher on Au(111),^69^ which is less hydrophilic) but the results are still of interest in comparing the LB/LS and the vesicle deposition techniques and the coverages as determined by AFM are comparable (*vide infra*).

### Depositions on gold

**Fig. 6:**
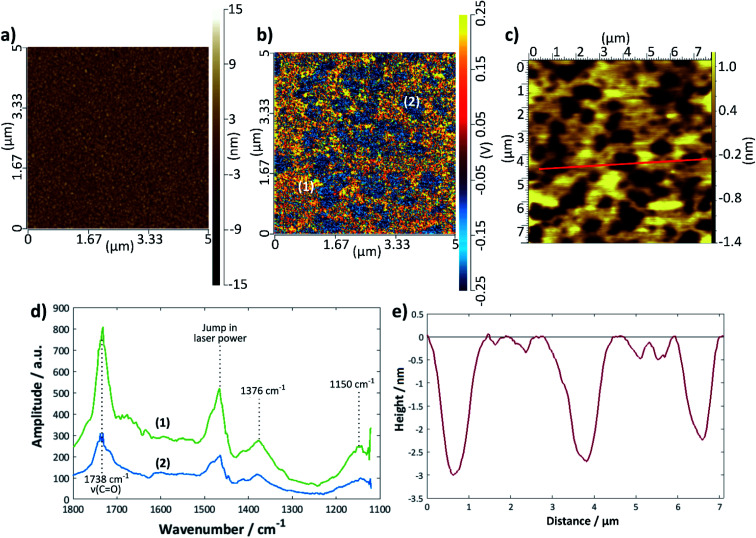
DMPC bilayer deposited on gold using the LB/LS technique at 16 °C: (a) contact mode topographical AFM image and (b) IR intensity map at 1738 cm^−1^ of the same area. (c) Tapping mode AFM image of same sample (measured previously), (d) IR spectra at points marked (1) and (2) on the image in (b). The jump in laser power around 1460 cm^−1^ is an instrumental artefact. (e) Height profile along the red line shown in (c).

Fig. 6 shows a set of representative results for a DMPC bilayer deposited on gold using the LB/LS technique. An image previously recorded using tapping-mode AFM is shown in Fig. 6c with a height profile given in Fig. 6e; the bilayer defects are clearly visible and relatively well-defined, although, as seen for the silicon samples, there is a variation in the exact depth measured. The IR spectra could only be measured in contact mode AFM, for which the topographical image showed no structural features (Fig. 6a); however, an IR intensity map of the ester C=O stretching mode at 1738 cm^−1^ (Fig. 6b) revealed defect structure very similar to that seen in the tapping mode images, confirming that the raised structure is indeed lipid. Individual spectra were measured across a 1100–1800 cm^−1^ range for points where there appeared to be good lipid bilayer coverage and also where there appeared to be a defect (Fig. 6d); the overall intensity of the latter was much lower than that of the former, as expected. It should be noted that the intensity may also be affected by the tilt angle of the lipids, although this effect is not yet fully understood for the AFM-IR technique; hence, areas of lower IR intensity may also be a reflection of lipids that are present but differently oriented with respect to the surface normal. Assuming that the surface selection rules apply as for reflection-absorption spectroscopy, more tilted or disordered chains would give rise to higher signal than upright or less tilted chains because in the latter case the transition dipole of the C=O stretching mode is oriented close to perpendicular to the chain axis. Therefore, it is likely that the blue regions on the sample correspond to defects where there is less lipid present. The decrease in signal is more commensurate with what may be expected for regions of monolayer than for slightly thinner regions with higher tilt angle. The peak at 1738 cm^−1^ may be assigned to a C=O stretch and that at 1376 cm^−1^ to a combination of a CH_2_ wagging mode with the methyl umbrella mode and C–C stretching modes^67,68^ (the scissoring modes usually seen around 1460 cm^−1^ are, unfortunately, hidden by the jump in laser power). The peak around 1150 cm^−1^ is likely to be the COC stretch of the ester groups;^68,69^ there may be a small shoulder around 1220 cm^−1^ arising from the antisymmetric P=O stretch but the intensity is too low for this to be confidently resolved. The C=O 1738 cm^−1^ peak was chosen for the intensity mapping as it was the most intense. This peak is actually composed of two peaks, one at ∼1728 cm^−1^, which is generally assigned to carbonyl groups participating in hydrogen bonding with water and one at ∼1740 cm^−1^, which is assigned to those not participating in hydrogen bonding with water.^70,71^ A band centre of 1738 cm^−1^ indicates relatively unsolvated bilayers, somewhere between the two states observed for DMPC on Au(111),^69^ but the shape of the band shows there is also a contribution from solvated groups, which suggests some water may be retained within the bilayer on deposition.

The extent of coverage, measured in the same way as for the silicon samples above, was found to be 66 (±3)% for the LB/LS technique using a water substrate, a slightly lower value than that seen for the same technique on a silicon surface. The average thickness measured across a large range of defects was found to be 4.1 (±0.3) nm. DMPC bilayer films on gold have been widely reported to have thicknesses of around 4.5 nm in the gel state (below *T*_m_), decreasing to around 3.5 nm in the liquid crystalline phase (above *T*_m_).^72^ The slightly lower value seen here is presumed to arise from a combination of surface roughness and a less than perfectly sharp AFM tip. Although the darker regions could, in principle, also result from phase co-existence, it is more likely that they correspond to defects because the temperature was always maintained 5–6 °C below the phase transition temperature and the IR signal is lower in these regions (*vide supra*).

**Fig. 7:**
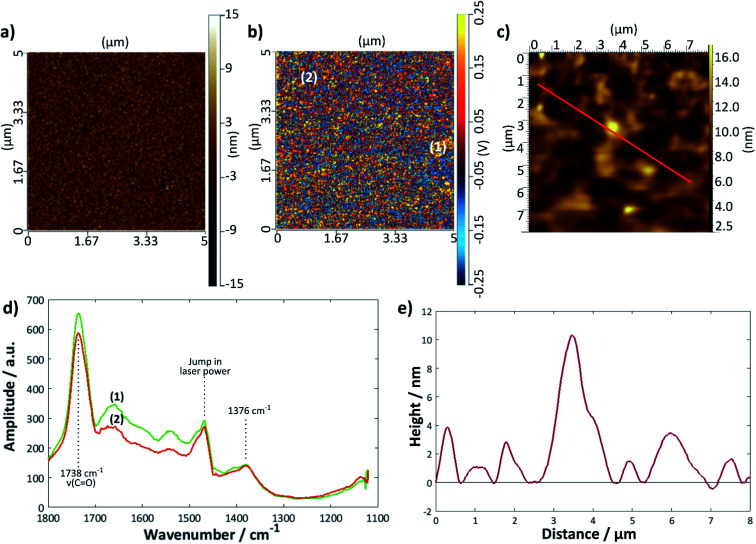
DMPC bilayer deposited on gold at 28 °C from vesicles: (a) contact mode topographical AFM image and (b) IR intensity map at 1738 cm^−1^ of the same area. (c) Tapping mode AFM image of same sample (measured previously), (d) IR spectra at points marked (1) and (2) on the image in (b). The jump in laser power around 1460 cm^−1^ is an instrumental artefact. (e) Height profile along the red line shown in (c).

A further gold sample was treated with the DMPC/UPW vesicle solution for 2 h > *T*_m_ (28 °C); a summary of the results is given in Fig. 7. The height profile (Fig. 7e) taken from the tapping mode AFM image shows a much rougher surface, with no obvious bilayer defects. Here, the AFM-IR images are helpful in determining the extent of bilayer coverage on the surface; the intensity map at 1738 cm^−1^ (Fig. 7b) shows a relatively uniform coverage compared with the bilayer deposited using the LB/LS technique, although in general there are fewer areas of high intensity—this may indicate that the surface lipid structure is less ordered overall, possibly with differently tilted or collapsed lipid bilayers. The IR spectra taken at two different points (Fig. 7d) showed similar peaks to that for the previous sample, with a slight difference in intensity between the two positions, although significantly less marked than for the LB/LS deposition sample.

## Conclusions

A variety of commonly-used techniques for depositing DMPC lipid bilayers onto silicon substrates have been compared using AFM to calculate coverages and AFM-IR to map the chemical groups at the surface; generally, vesicle fusion was found to be considerably more effective than LB/LS, with almost perfect coverage (∼99%) seen after 60 s in all cases when sonicated >*T*_m_. However, when the silicon surfaces were exposed to the vesicles for longer times, rougher surfaces were observed as well as some instances of multilayer adsorption. The bilayer was much slower to form at lower temperatures, presumably a result of weaker interactions between adsorbed vesicles and hence lower probability of vesicle rupture. LB/LS allowed a more controlled deposition process but the inclusion of cholesterol was necessary to obtain coverages >90%. AFM-IR was used to confirm the presence of the lipid on gold surfaces for both LB/LS deposition below *T*_m_ and vesicle deposition above *T*_m_, although it was unable to detect the lipids on silicon because of the lower thermal expansion coefficient. Hence AFM-IR was able to confirm the suitability of AFM to calculate surface coverage.

The quantitative approach towards comparing methods of lipid bilayer deposition outlined here permits a straightforward protocol for determining the optimum technique for creating model cell membranes that is applicable across a range of materials and systems. Understanding this gateway to the cell is a key area of interest in many fields of research and hence the construction of a viable model bilayer is of critical importance.

## Conflicts of interest

There are no conflicts of interest to declare.

## Supplementary Material

RA-011-D1RA01920A-s001
