# Supplementary material for: A quantitative determination of lipid bilayer deposition efficiency using AFM
Source: RSC Adv. 2021 Jun 2;11(32):19768–78. doi: 10.1039/d1ra01920a (PMC9033767; doi:10.1039/d1ra01920a)
Supplement: RA-011-D1RA01920A-s001 [file RA-011-D1RA01920A-s001.pdf]

Electronic Supplementary Material (ESI) for RSC Advances.
This journal is © The Royal Society of Chemistry 2021

A quantitative determination of lipid bilayer deposition efficiency using AFM

Mary H. Wood,[1*†] David C. Milan,[2] Richard J. Nichols,[2] Michael T. L. Casford[3] and Sarah L. Horswell[1*]

[1]*School of Chemistry, University of Birmingham, Birmingham, UK, B15 2TT*

[2]*Department of Chemistry, University of Liverpool, Liverpool, UK, L69 7ZD*

[3]*Department of Chemistry, University of Cambridge, Lensfield Road, Cambridge, CB2 1EW*

[†]*Present address: Laboratory of Nanobiotechnology, EPFL, 1015 Lausanne, Switzerland*

** mary.wood@epfl.ch, s.l.horswell@bham.ac.uk*

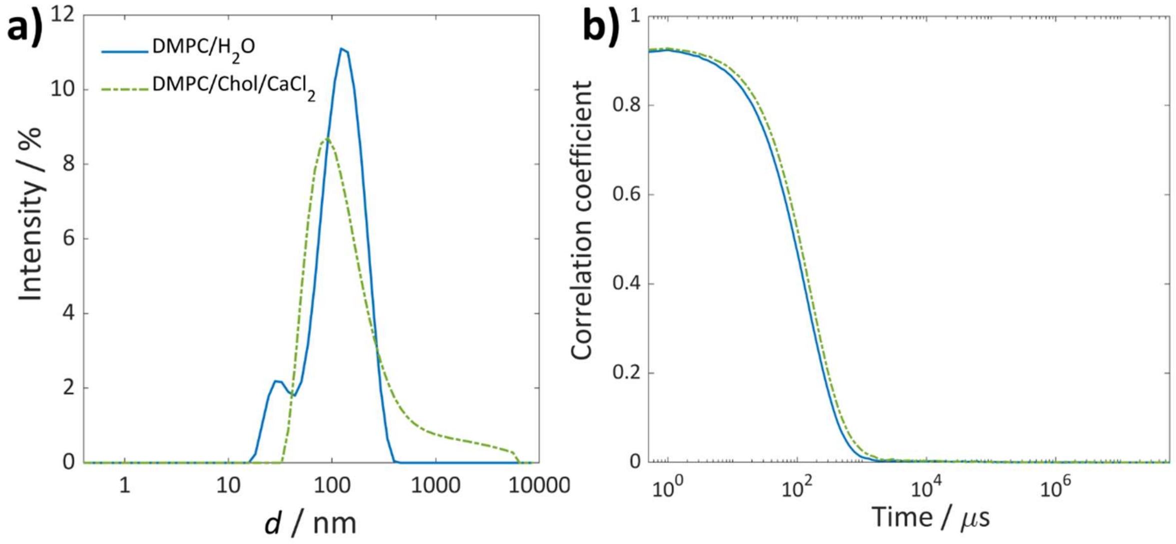

Figure S1. (a) Example DLS size intensity plots and (b) corresponding correlograms for DMPC/H_2O vesicles (blue) and DMPC/Chol/$CaCl_2$(*aq*) vesicles (green, dashed). Z-average diameters were 108 nm and 113 nm and PDIs 0.27 and 0.33, respectively.

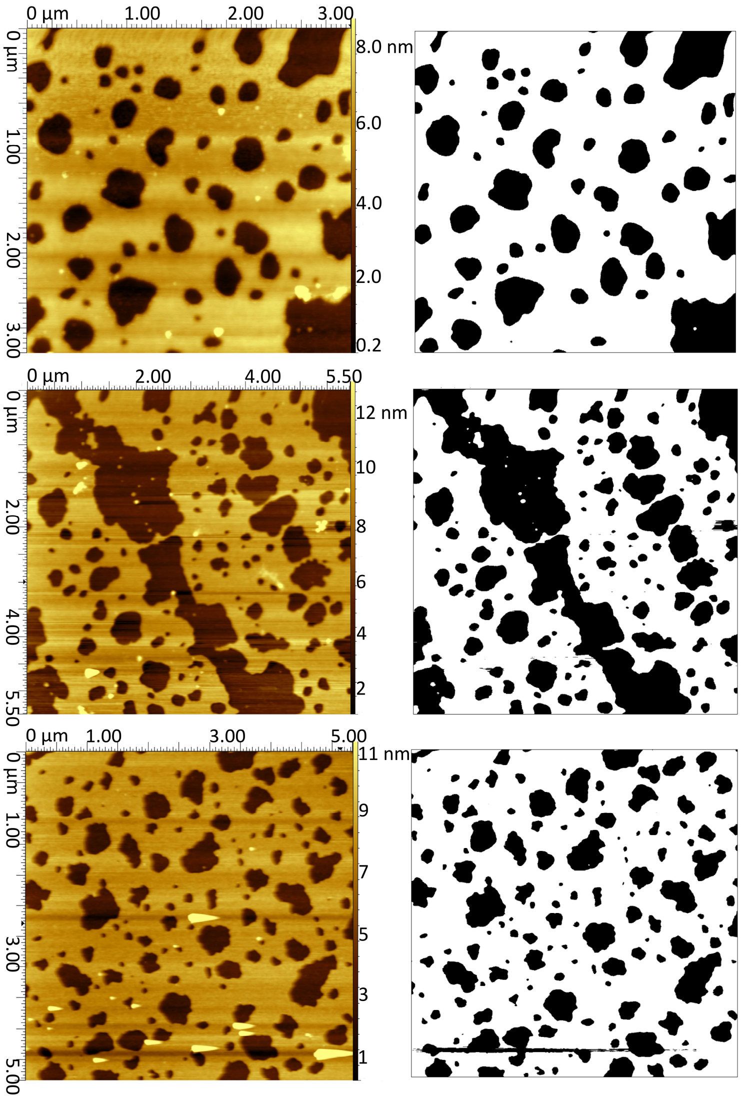

Figure S2. Further examples of AFM topographical images (left) and corresponding binarised data (right) for LB/LS bilayers of DMPC deposited onto silicon from UPW at 16 °C.

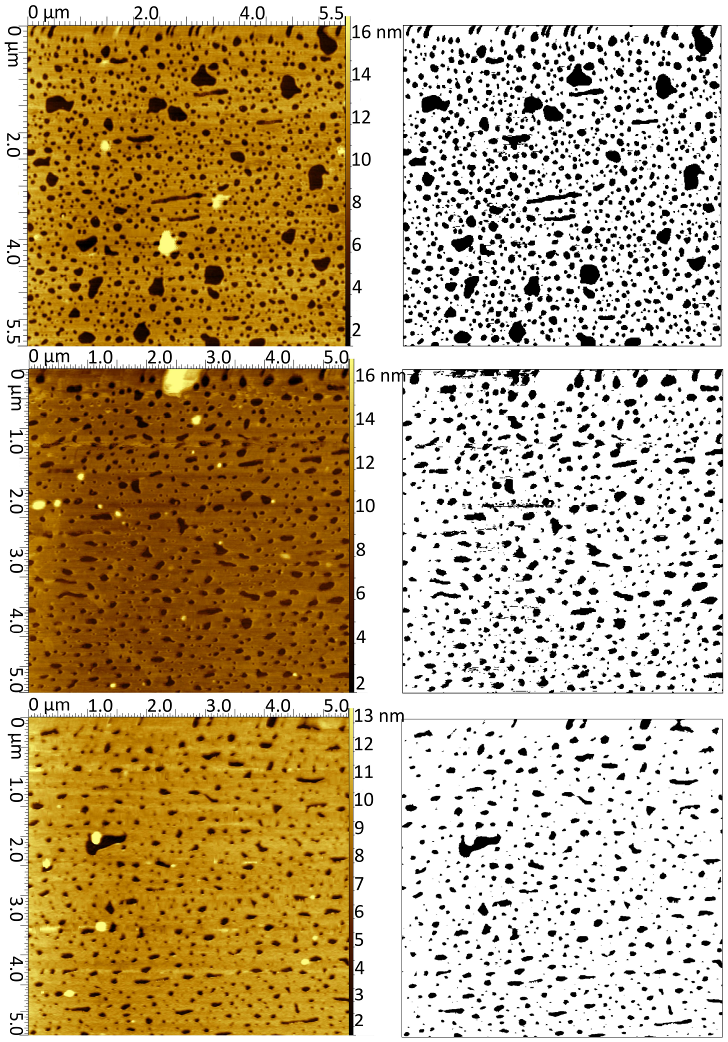

Figure S3. Further examples of AFM topographical images (left) and corresponding binarised data (right) for LB/LS bilayers of DMPC deposited onto silicon from $CaCl_2$ (0.1 M) at 16 °C.

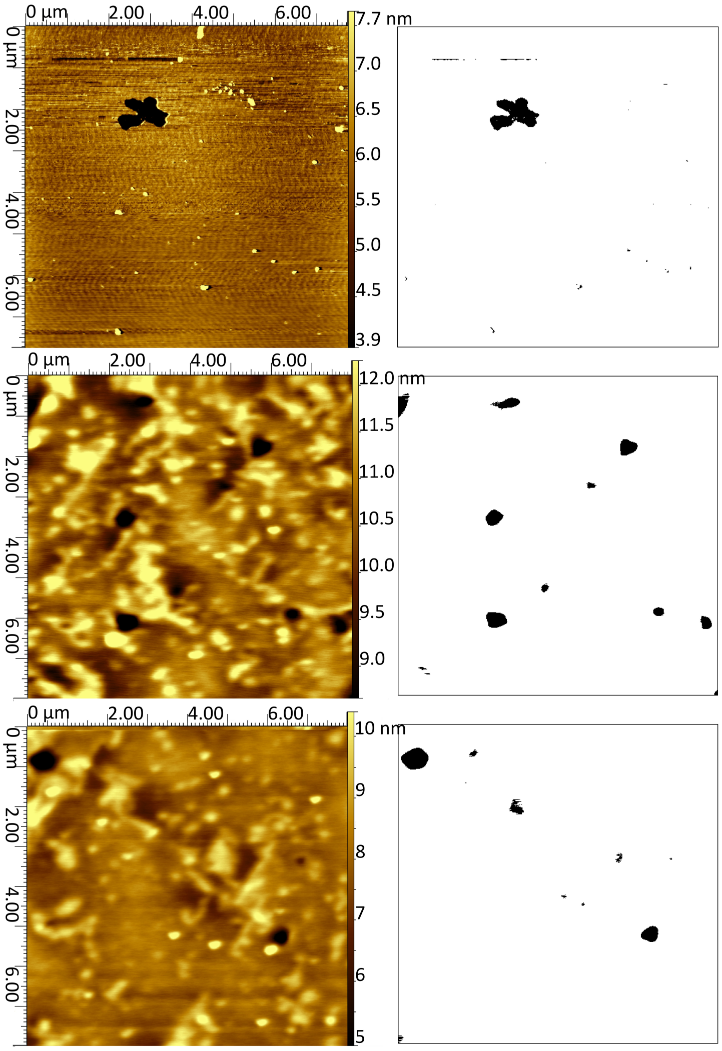

Figure S4. Further examples of AFM topographical images (left) and corresponding binarised data (right) for LB/LS bilayers of DMPC:cholesterol (3:1) deposited onto silicon from UPW at 16 °C.

To determine the layer thickness values reported in Table 1, a large number of height profiles, such as those in Figure S5, were taken across each AFM image, the background was subtracted and the depths of each defect were recorded to give an overall average. For example, Figure S5 shows two height profiles taken across images for a DMPC LB/LS bilayer on silicon; obvious outliers were omitted from the bilayer calculation (anything < 3.5 nm was discounted as being less than a full defect) but there is still a relatively large range of error even for one image. For example, in some cases, it appears that the second layer has not adhered.

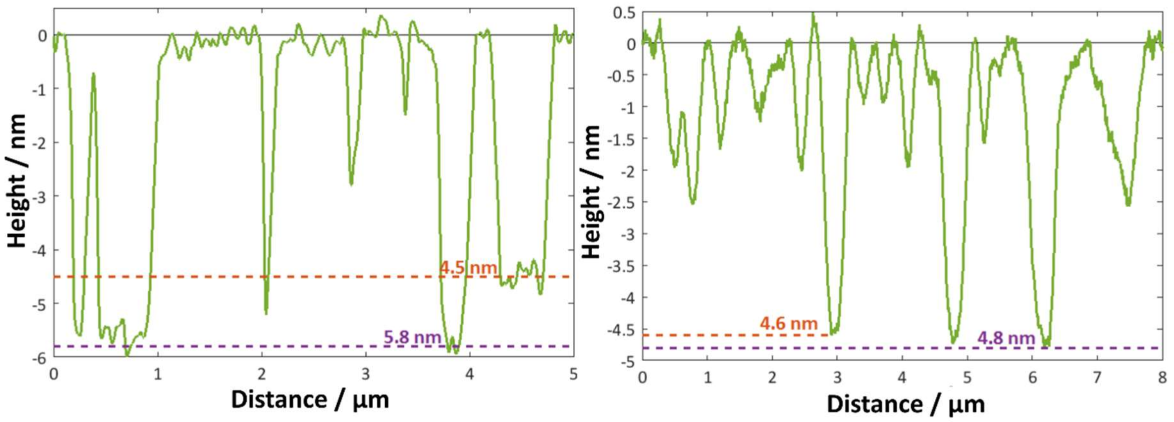

Figure S5. Two examples of height profiles taken across AFM images for a DMPC bilayer deposited on silicon *via* LB/LS from a water subphase (at 16°C) showing the range of defect depths.

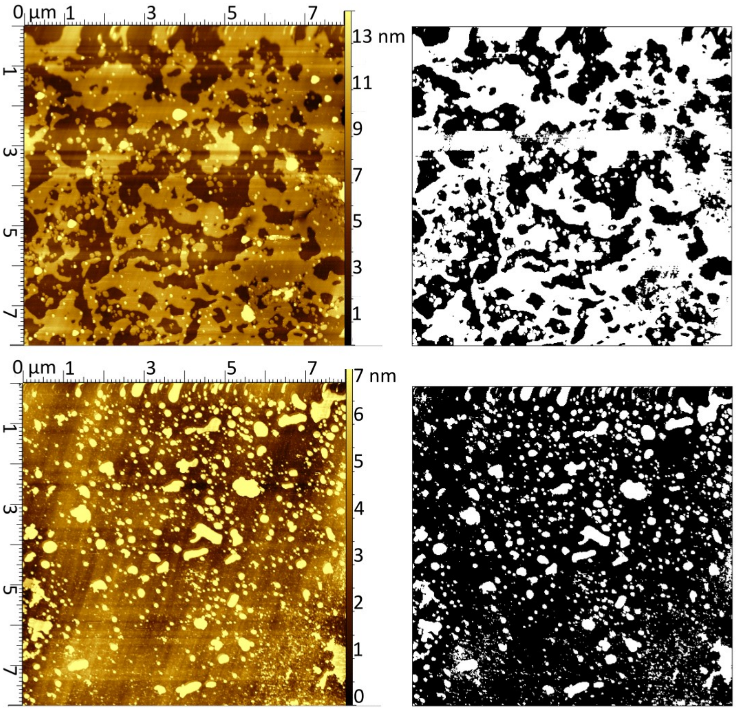

Figure S6. Further examples of AFM topographical images (left) and corresponding binarised data (right) for bilayers of DMPC deposited onto silicon from vesicles in UPW at 28 °C; substrate removed after 30 s.

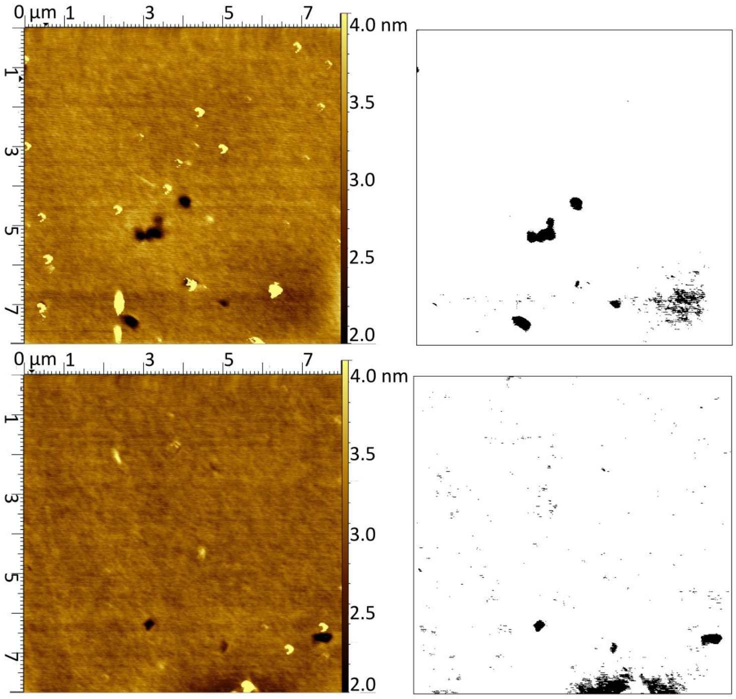

Figure S7. Further examples of AFM topographical images (left) and corresponding binarised data (right) for bilayers of DMPC deposited onto silicon from vesicles in UPW at 28 °C; substrate removed after 60 s.

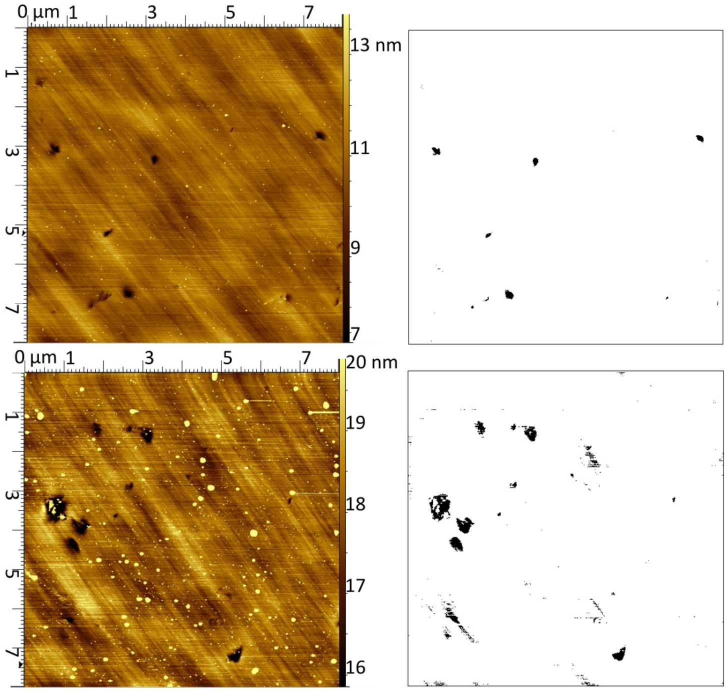

Figure S8. Further examples of AFM topographical images (left) and corresponding binarised data (right) for bilayers of DMPC deposited onto silicon from vesicles in UPW at 28 °C; substrate removed after 300 s.

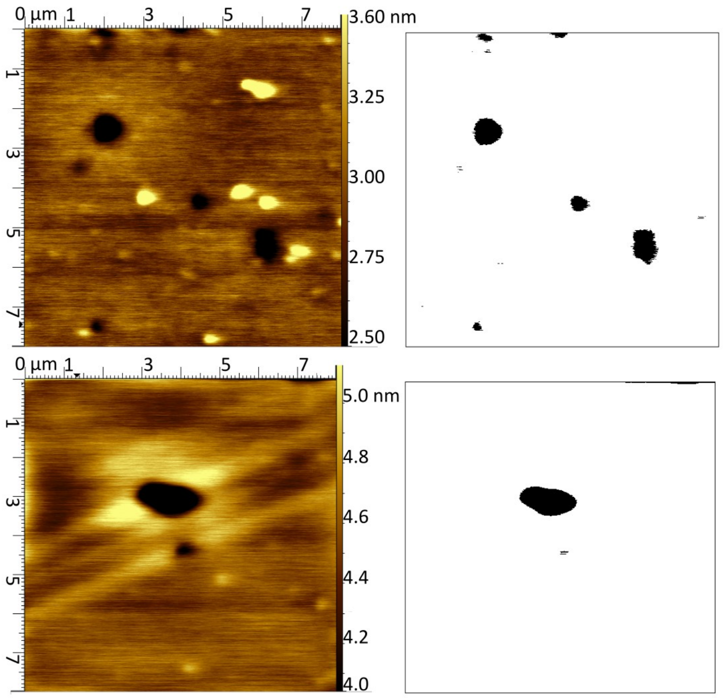

Figure S9. Further examples of AFM topographical images (left) and corresponding binarised data (right) for bilayers of DMPC deposited onto silicon from vesicles in UPW at 28 °C; substrate removed after 1800 s.

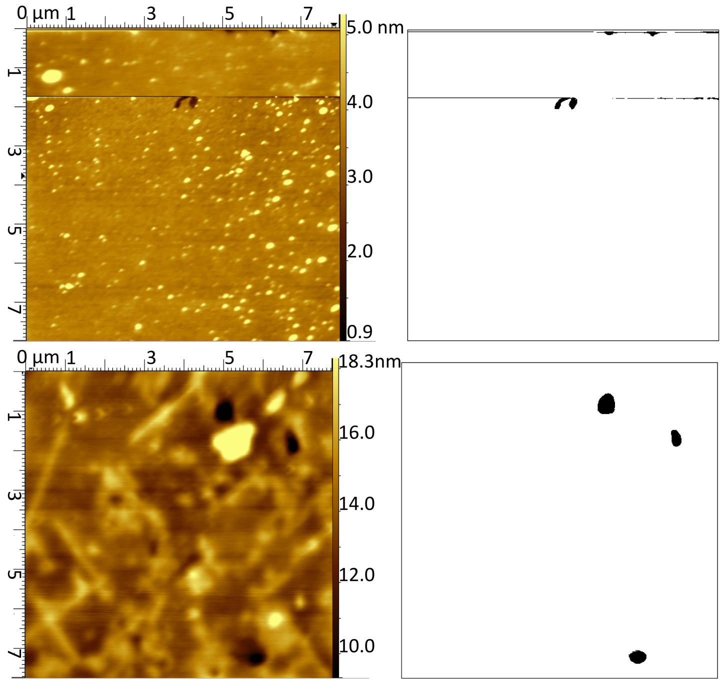

Figure S10. Further examples of AFM topographical images (left) and corresponding binarised data (right) for bilayers of DMPC deposited onto silicon from vesicles in UPW at 28 °C; substrate removed after 7200 s.

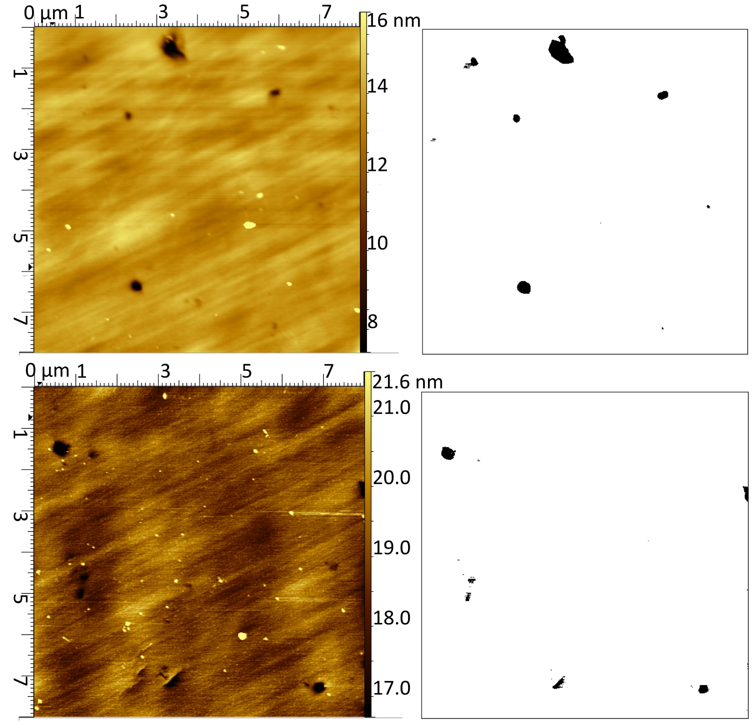

Figure S11. Further examples of AFM topographical images (left) and corresponding binarised data (right) for bilayers of DMPC deposited onto silicon from vesicles in $CaCl_2$ (0.1 M) at 28 °C; substrate removed after 30 s.

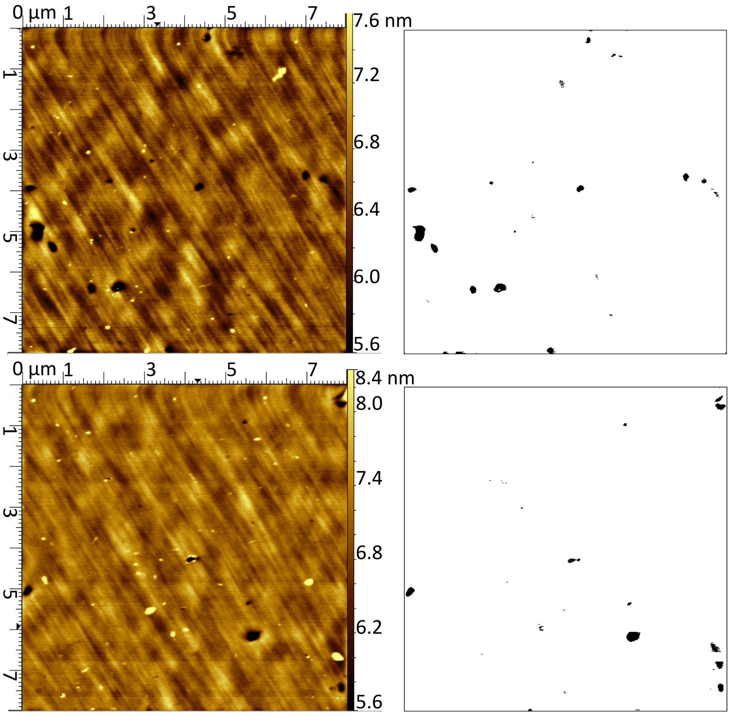

Figure S12. Further examples of AFM topographical images (left) and corresponding binarised data (right) for bilayers of DMPC deposited onto silicon from vesicles in $CaCl_2$ (0.1 M) at 28 °C; substrate removed after 60 s.

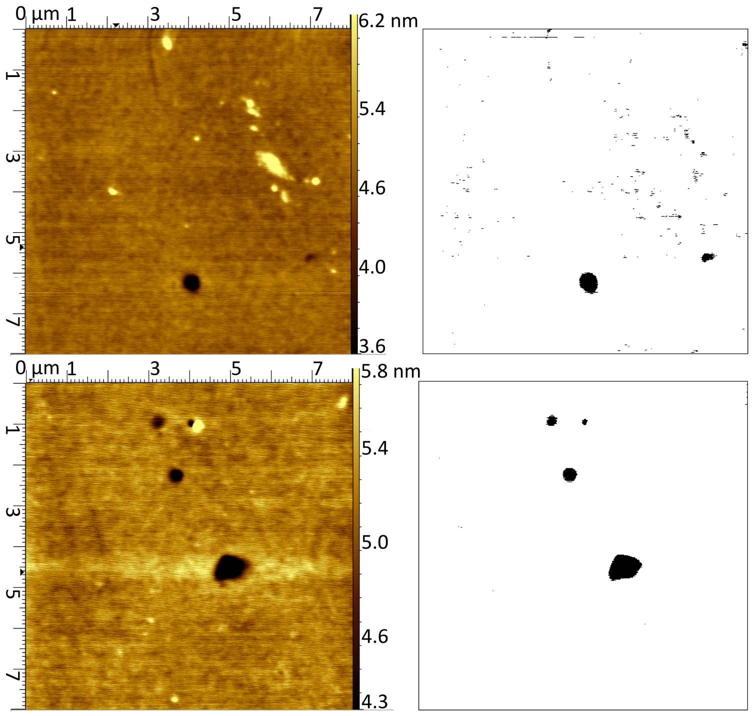

Figure S13. Further examples of AFM topographical images (left) and corresponding binarised data (right) for bilayers of DMPC deposited onto silicon from vesicles in $CaCl_2$ (0.1 M) at 28 °C; substrate removed after 120 s.

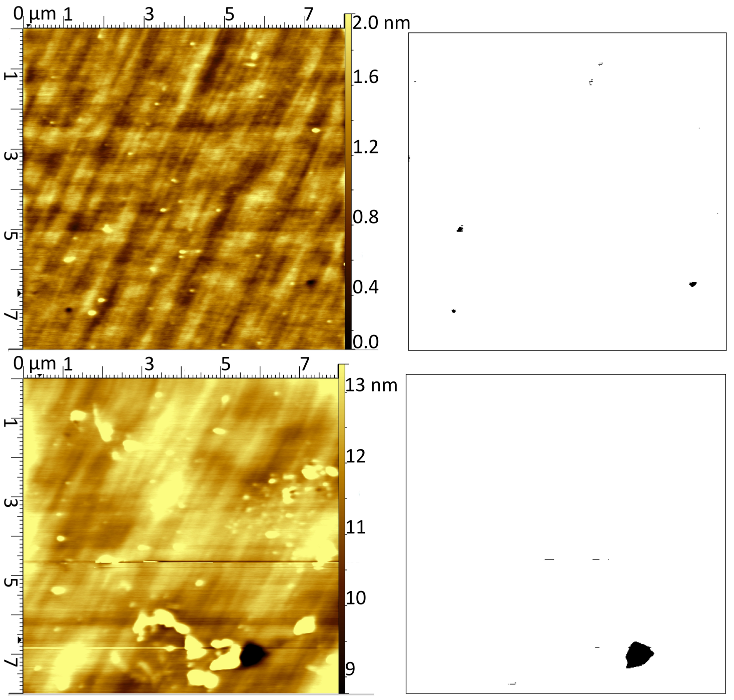

Figure S14. Further examples of AFM topographical images (left) and corresponding binarised data (right) for bilayers of DMPC deposited onto silicon from vesicles in $CaCl_2$ (0.1 M) at 28 °C; substrate removed after 1800 s.

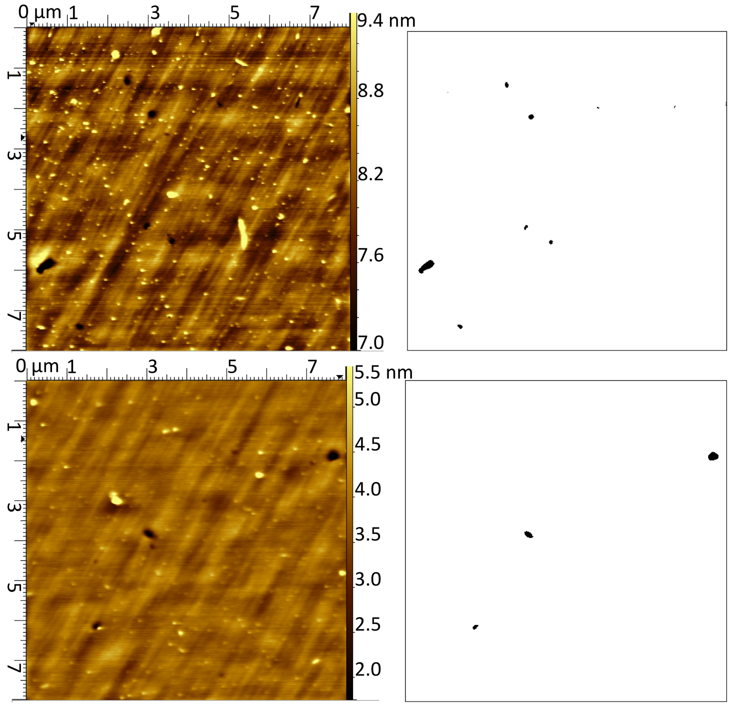

Figure S15. Further examples of AFM topographical images (left) and corresponding binarised data (right) for bilayers of DMPC deposited onto silicon from vesicles in $CaCl_2$ (0.1 M) at 28 °C; substrate removed after 7200 s.

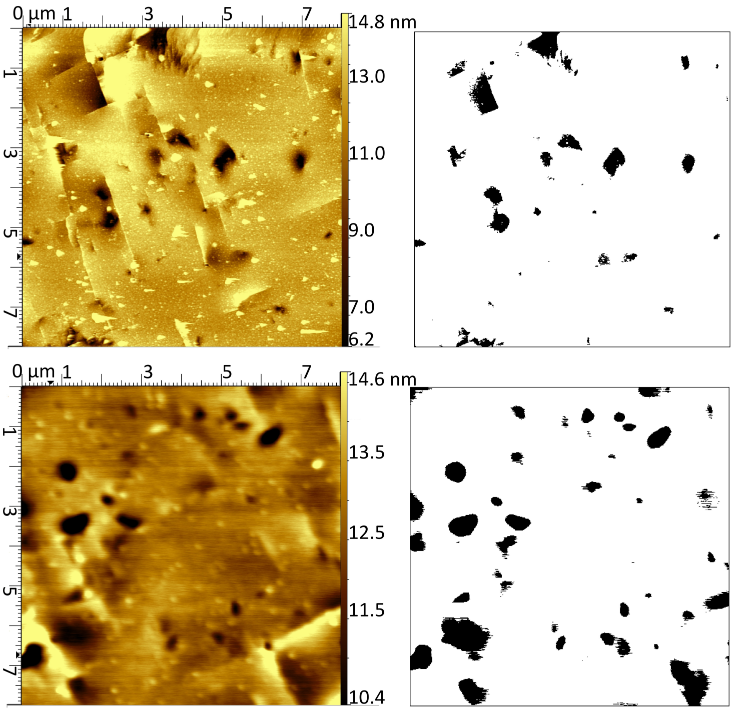

Figure S16. Further examples of AFM topographical images (left) and corresponding binarised data (right) for bilayers of DMPC/cholesterol (3:1) deposited onto silicon from vesicles in UPW at 28 °C; substrate removed after 30 s.

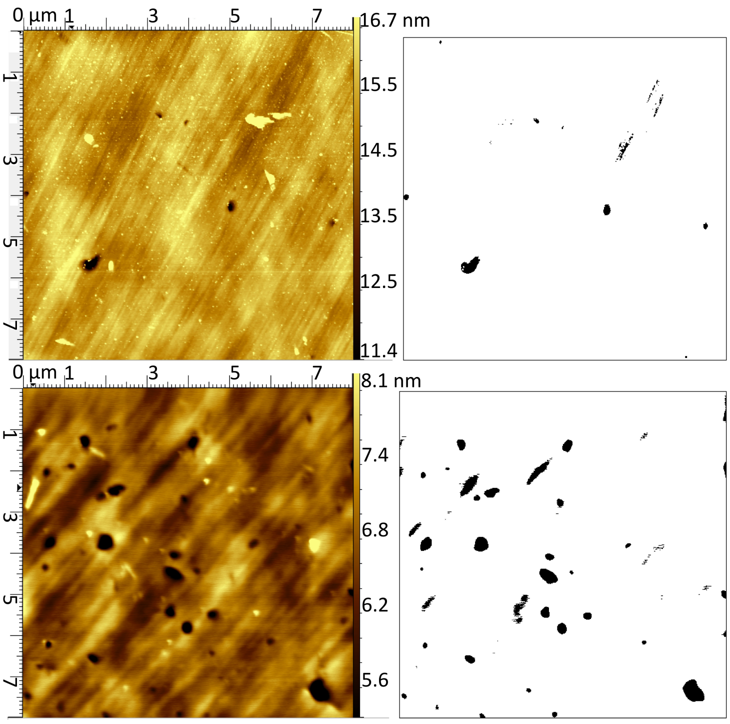

Figure S17. Further examples of AFM topographical images (left) and corresponding binarised data (right) for bilayers of DMPC/cholesterol (3:1) deposited onto silicon from vesicles in UPW at 28 °C; substrate removed after 60 s.

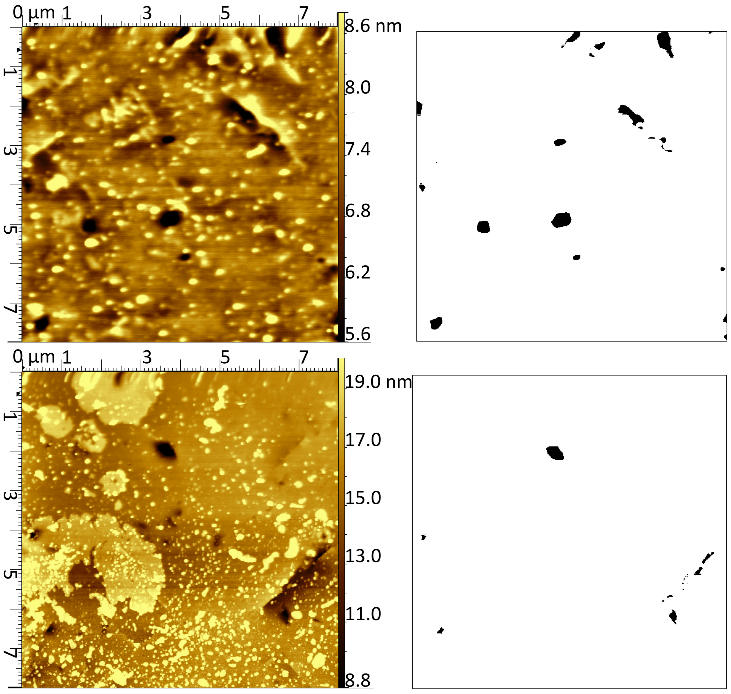

Figure S18. Further examples of AFM topographical images (left) and corresponding binarised data (right) for bilayers of DMPC/cholesterol (3:1) deposited onto silicon from vesicles in UPW at 28 °C; substrate removed after 120 s.

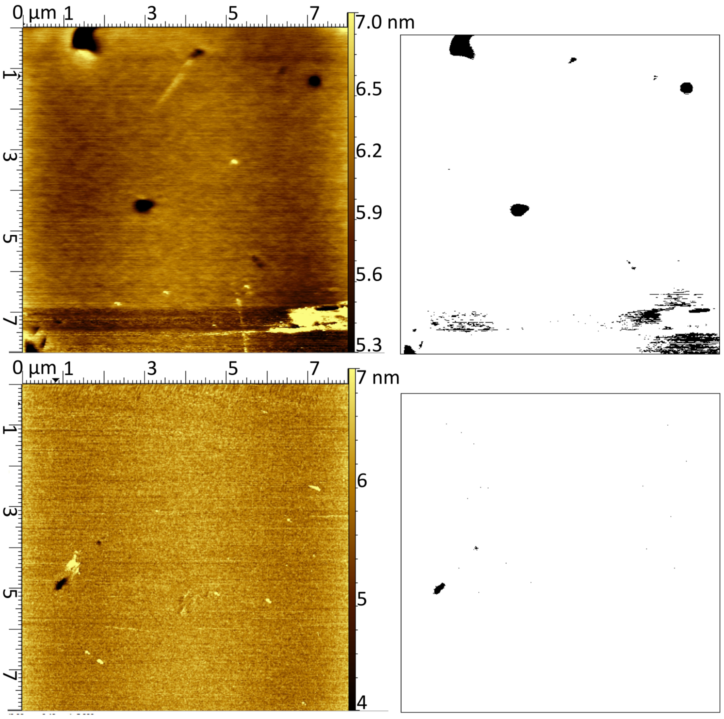

Figure S19. Further examples of AFM topographical images (left) and corresponding binarised data (right) for bilayers of DMPC/cholesterol (3:1) deposited onto silicon from vesicles in UPW at 28 °C; substrate removed after 600 s.

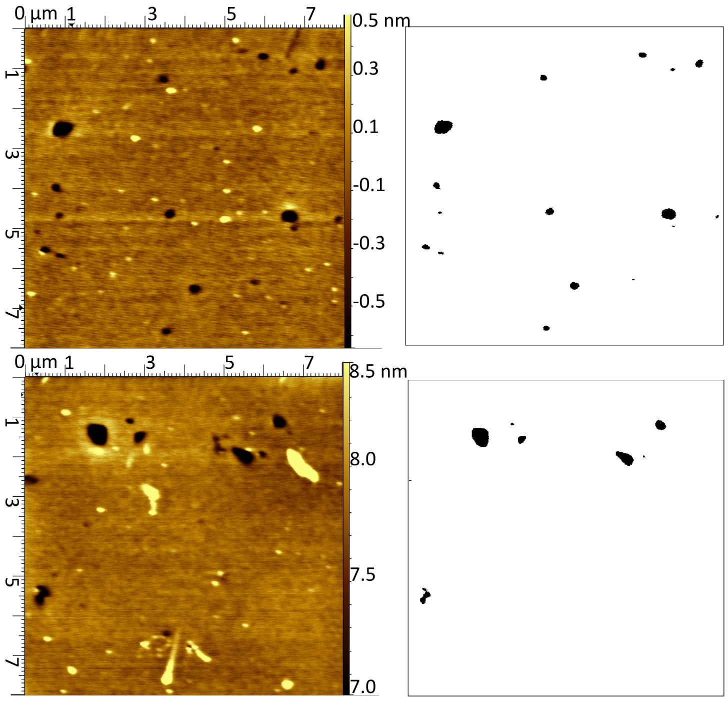

Figure S20. Further examples of AFM topographical images (left) and corresponding binarised data (right) for bilayers of DMPC/cholesterol (3:1) deposited onto silicon from vesicles in UPW at 28 °C; substrate removed after 1200 s.

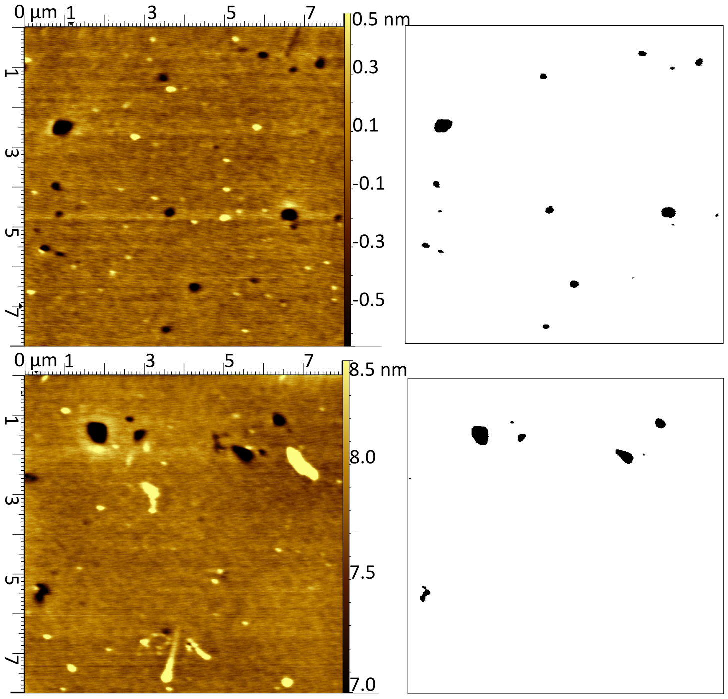

Figure S21. Further examples of AFM topographical images (left) and corresponding binarised data (right) for bilayers of DMPC/cholesterol (3:1) deposited onto silicon from vesicles in UPW at 28 °C; substrate removed after 1800 s.

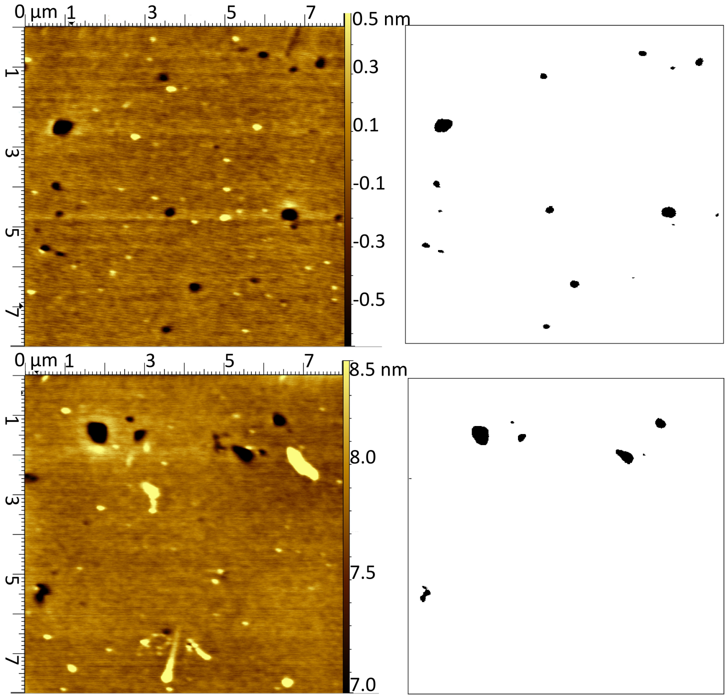

Figure S22. Further examples of AFM topographical images (left) and corresponding binarised data (right) for bilayers of DMPC/cholesterol (3:1) deposited onto silicon from vesicles in UPW at 28 °C; substrate removed after 7200 s.

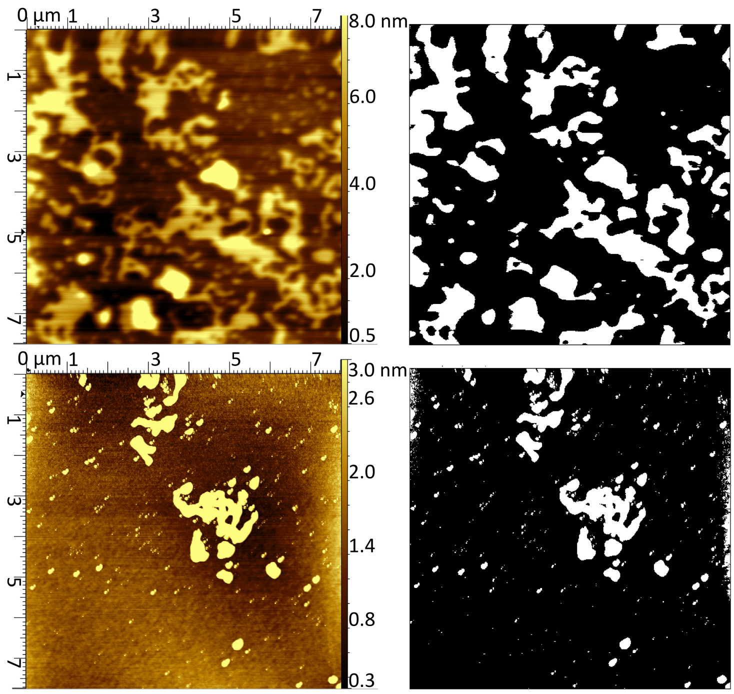

Figure S23. Further examples of AFM topographical images (left) and corresponding binarised data (right) for bilayers of DMPC deposited onto silicon from vesicles in UPW at 16 °C; substrate removed after 60 s.

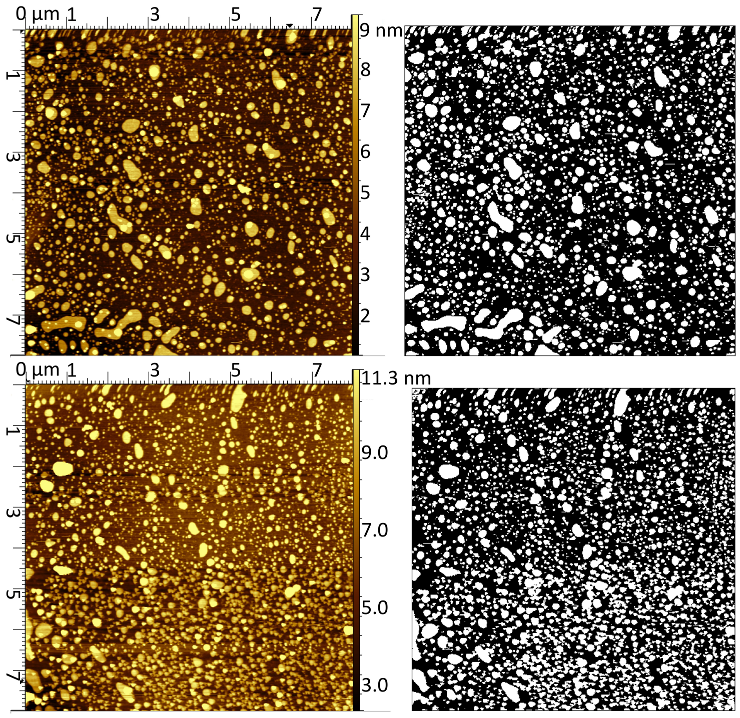

Figure S24. Further examples of AFM topographical images (left) and corresponding binarised data (right) for bilayers of DMPC deposited onto silicon from vesicles in UPW at 16 °C; substrate removed after 300 s.

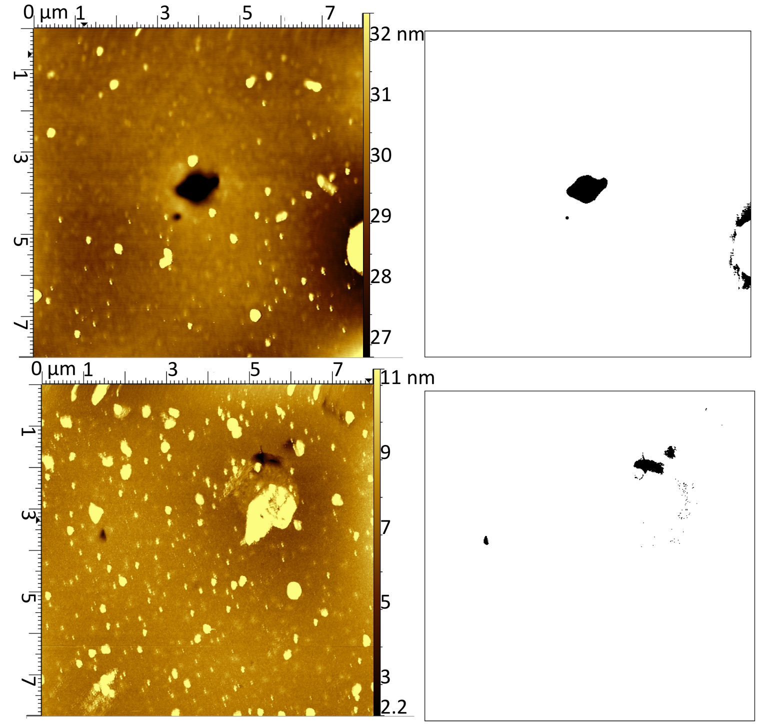

Figure S25. Further examples of AFM topographical images (left) and corresponding binarised data (right) for bilayers of DMPC deposited onto silicon from vesicles in UPW at 16 °C; substrate removed after 1800 s.

Note that samples were analysed as soon as possible after the deposition process, to minimise any possible effect of change over time. Nevertheless, a few samples were re-analysed after some months (with no special storage) and showed no change in overall average surface coverage.
